# Non-weight-bearing exercise attenuates papain-induced knee osteoarthritis in rats via the TLR4/MyD88/NF-κB signaling pathway

**DOI:** 10.1186/s13018-023-04201-w

**Published:** 2023-09-18

**Authors:** Kewen Wang, Xianji Zhang, Xin Li, Dekun Li, Ziliang Shan, Changfeng Yao

**Affiliations:** College of Acupuncture and Massage, Anhui University of Chinese Medicine, Hefei, 230012 Anhui China

**Keywords:** Chondrocytes, Knee osteoarthritis, Non-weight-bearing exercise, TLR4/MyD88/NF-κB signaling pathway

## Abstract

**Background and Aim:**

Knee osteoarthritis (KOA) is characterized by joint wear and degeneration. Unfortunately, the medical community currently lacks effective treatment options for this disease. Suspension exercise therapy is considered an effective form of non-weight-bearing exercise for treating KOA. However, its mechanism of intervention in KOA is unclear. Therefore, this study aimed to evaluate the protective effects of non-weight-bearing exercise on rats with KOA and attempted to explore the underlying mechanisms.

**Methods:**

In this study, a papain-induced KOA model was constructed, and the pathological changes in cartilage tissue were observed by hematoxylin and eosin (H&E) staining and scored according to the Mankin scoring principle. The serum levels of interleukin (IL)-1β, IL-6, and tumor necrosis factor-α (TNF-α) were detected by enzyme-linked immunosorbent assay. Reverse transcription–quantitative polymerase chain reaction and Western blotting were used to detect the expression of mRNA and proteins in the TLR4/MyD88/NF-κB signaling pathway.

**Results:**

H&E staining and Mankin score data confirmed that non-weight-bearing exercise significantly improved articular cartilage degradation compared with that in the model group. Further, we observed that non-weight-bearing exercise differentially reduced serum levels of IL-1β, IL-6, and TNF-α. Mechanistically, non-weight-bearing exercise downregulated gene and protein expression of TLR4, MyD88, and NF-κB in cartilage tissue.

**Conclusion:**

Non-weight-bearing exercise resulted in the progression of KOA by modulating the TLR4/MyD88/NF-κB signaling pathway and decreasing the levels of the inflammatory cytokines IL-1β, IL-6, and TNF-α to slow down the degeneration of articular cartilage.

## Introduction

Knee osteoarthritis (KOA) is a prevalent degenerative joint disease that primarily affects the articular cartilage, leading to inflammation-induced damage and the development of bone osteophytes. Clinical symptoms include knee swelling, joint pain, stiffness, and restricted motion. The prevalence of KOA increases with age and is more predominant in women than in men [1]. This disease adversely impacts the quality of life due to chronic pain and limited physical function, leading to significant medical expenses and psychological burden [2].

Non-weight-bearing exercises can mitigate damage caused by uneven pressure on the knee joint during weight-bearing activities. These exercises also help reduce inflammation, strengthen exercise muscles and ligaments associated with the knee joint, and promote functional recovery [3]. Suspension exercise therapy, a popular sports rehabilitation technology, involves non-weight-bearing training. It utilizes suspension devices to eliminate the impact of gravity on the human body, creating an unstable movement environment. Such an unstable environment promotes muscle contractions beneficial for sustained improvements in muscle strength [4]. The quadriceps and hamstring muscles maintain knee joint function and stability. Patients can enhance their knee joint stability through active contraction during exercise [5]. However, the mechanism by which non-weight-bearing exercise intervenes in KOA is unclear.

Numerous studies indicate that inflammatory factors play a crucial role in developing KOA. These factors contribute to knee joint metabolism and maintaining homeostasis within the joint. Specifically, inflammatory cartilage tissue releases cytokines such as interleukin (IL)-1β, IL-6, and tumor necrosis factor-α (TNF-α), which are responsible for cartilage matrix degradation and bone destruction in osteoarthritis [6]. The TLR4/myeloid differentiation factor 88 (MyD88)/NF-κB signaling pathway is a MyD88-dependent pathway for MyD88. TLR4 is a transmembrane protein widely present on the surface of animal cell membranes. It belongs to the group of 10 Toll-like receptors (TLRs) [7]. TLR4 can trigger the production of several inflammatory factors. It facilitates the release of inflammatory mediators by detecting both damage-associated molecule patterns and pathogen-associated molecule patterns [8, 9]. MyD88 is a soluble adaptor protein located in the cytoplasm of animal cells. It is instrumental in receiving immune signals from TLRs (including TLR4), translating and integrating them, and subsequently transmitting them to downstream signaling pathways. Therefore, it serves as a significant hub of immune signal transduction [10]. NF-κB is a dimerized protein residing in the cytoplasm of animal cells. Upon activation by immune signals transmitted by the TLR4/MyD88 signaling pathway, NF-κB is activated, phosphorylated, and translocated to the nucleus, regulating the transcription and expression of genes involved in immune and inflammatory responses [11]. However, how the TLR4/MyD88/NF-κB signaling pathway affects KOA and its regulatory mechanisms have not been extensively studied.

Hence, this study examined the impact of non-weight-bearing exercise on TNF-α, IL-1β, and IL-6 levels, as well as the TLR4/MyD88/NF-κB signaling pathway, in a rat model of KOA to provide further scientific evidence for the clinical use of non-weight-bearing exercise in treating KOA.

## Materials and methods

### Experimental animals and groupings

All animal experiments were carried out by the "Guiding Opinions on the Good Treatment of Laboratory Animals" issued by the Ministry of Science and Technology of China and approved by the Experimental Animal Ethics Committee of Anhui University of Traditional Chinese Medicine (approval number: AHUCM-rats-2023027). Forty male SD rats (300 ± 10 g, 8 weeks old) were procured from Pengyue Experimental Animal Breeding Co. (Jinan, China). The rats were housed in separate cages under standard conditions, including a room temperature of 24 °C, a standard chow diet, and ad libitum access to food. The rats were randomly assigned to the following five groups, with eight rats in each group: (1) control group, (2) KOA group (injected with papain), (3) group subjected to suspension exercise 1 (Se1), (4) group subjected to suspension exercise 2 (Se2), and (5) group subjected to suspension exercise 3 (Se3).

### Establishment of KOA animal model

A rat model of KOA was established by the intra-articular injection of 4% papain (Biosharp, Hefei, China). The rat was anesthetized by injecting 30 mg/kg of 3% sodium pentobarbital into the abdominal cavity. The villi were shaved off, and the right knee was sterilized. Then, 0.2 mL of 4% papain solution was injected into the joint cavity. This procedure was repeated three times on days 1, 4, and 7 [12].

### Intervention method

Three groups of rats in the treatment group were suspended by their hind limbs. This hanging technique was based on the previous tail suspension method [13] and improved using traction ropes and back straps to fix the rat's hip joints, significantly reducing the pain caused by the tail suspension. The specific steps followed were as follows: the back strap passed through the rat's lower limbs, wrapped around the hip joints, and exposed the knee joints. The tightness should be moderate. The traction rope was tied to the iliac bone of the rat and adjusted with a loose buckle for fixation. The other end of the traction rope was tied to the top of the cage. The angle between the rat's trunk and the horizontal plane was adjusted to ensure free movement of the rat's forelimbs, and the hind limbs were suspended. The rat, when startled, could involuntarily perform active non-weight-bearing flexion and extension exercises of the hind limbs by tapping the cage on time, thereby simulating the non-weight-bearing exercise of the knee joint performed clinically. The suspension exercise was performed 1 week after inducing KOA. The first group of suspension exercises was performed once a day for 10 min. The second group of suspension exercises was performed twice a day for 10 min each time. Finally, the third group of suspension exercises was performed thrice a day for 10 min each time. All groups continued these exercises for 4 weeks, except on Saturdays and Sundays. No intervention was made in the control and model groups.

### Sample processing

Forty rats were anesthetized with sodium pentobarbital after 4 weeks of intervention, and their abdominal aortas were dissected. Then, the abdominal aorta blood of the rats was collected and kept at room temperature for 20 min. The blood was coagulated and centrifuged at 3000 rpm and 4 °C for 15 min using a tabletop centrifuge. Then, the serum was separated and tested for the expression of serum levels of IL-1β, IL-6, and TNF-α using ELISA. Further, the knee cartilage tissues from the right distal femur and tibial plateau of rats were removed on an ice box, placed in cryogenic tubes, and stored at − 80 °C for extracting study-related proteins and mRNA, and partially used for hematoxylin and eosin (H&E) staining observation and Mankin scoring.

### H&E staining and Mankin score

The knee tissues from four rats in each group were fixed with 4% paraformaldehyde for 24 h. Paraffin specimens were prepared by decalcifying the tissues with EDTA, dehydrating them with gradient ethanol, removing them with xylene, and embedding them with conventional paraffin. The specimens were cut into sections of about 3–5 μm thickness and stained with H&E. The staining of the knee cartilage sections was observed under a light microscope (Olympus, Japan) with a magnification of 200 times. After H&E staining, two experienced observers blinded to the experimental conditions scored the sections using the Mankin scoring principle [14]. This helped evaluate the severity of cartilage structure, chondrocytes, matrix staining, and tidal line damage on a scale of 0–14 (Table 1).

**Table 1:** Mankin scoring criteria

Category	Score
*Structure*	
Normal	0
Surface irregularities	1
Pannus and surface	2
Clefts to transitional zone	3
Clefts to radial zone	4
Clefts to calcified zone	5
Complete disorganizations	6
*Cells*	
Normal	0
Diffuse hypercellularity	1
Clusters	2
Hypocellularity	3
*Matrix staining*	
Normal	0
Slight reduction	1
Moderate reduction	2
Severe reduction	3
No staining	4
*Tidemark integrity*	
Intact	0
Destroyed	1

### ELISA

First, blood was collected from the abdominal aorta of eight rats in each group and kept at room temperature for 20 min. The blood was coagulated and then centrifuged at 3000 rpm and 4 °C for 15 min using a benchtop centrifuge (JW3021HR; Anhui Jiawen Instrument and Equipment Co., Ltd., China) to isolate the serum. Then, the expression of serum levels of IL-1β, IL-6, and TNF-α was detected using ELISA kits (JYM0419Ra, JYM0646Ra, and JYM0635Ra; Wuhan Genome Technology Co., Ltd, China), and the experiments were carried out following the manufacturer’s protocols.

### RNA extraction and RT-qPCR

Articular cartilage tissue samples were used to extract total RNA using the TRIzol reagent (Life Technologies, CA, USA). A PrimeScript RT reagent Kit (TaKaRa, Dalian, China) was used for obtaining cDNA and stored at − 80 °C for further testing. Fluorescent qPCR was performed using SYBR (Novoprotein, Shanghai, China), and the results were calculated using the 2^−ΔΔCt^ method. The primer sequences were as follows: β-actin forward: CCCATCTATGAGGGTTACGC; β-actin reverse: TTTAATGTCACGCACGATTTC; TLR4 forward: TAGCCATTGCTGCCAACATC; TLR4 reverse: ACACCAACGGCTCTGGATAA; MyD88 forward: GCATGGTGGTGGTTGTTTCT; MyD88 reverse: TCTGTTGGACACCTGGAGAC; NF-κBp65 forward: TCTACAGGCAGAAGGCGGAGGA; NF-kBp65 reverse: TGGCGTCTGACACCACAGGTTC.

### Western blot analysis

The cartilage tissue samples were collected and lysed by adding RIPA cell lysis solution (Beyotime, Shanghai, China). The supernatant was collected by centrifugation. The gels were then prepared, electrophoresed, and transferred to PVDF membranes (Millipore, MA, USA) before being poured into a closure solution and left for 2 h. Primary antibodies were diluted with closure solution as follows: TLR4 rabbit antibody (Bioss, Beijing, China), 1:1500; MyD88 mouse antibody (Santa Cruz, CA, USA), 1:1000; NF-κB p65 mouse antibody (Proteintech, IL, USA), 1:2000; p-NF-κB p65 rabbit antibody (CST, MA, USA); 1:1000. The PVDF membranes were incubated at 4 °C for 12 h. After completion of this step, a washing solution PBST (Zs-BIO, Beijing, China) was added. The horseradish peroxidase–labeled secondary antibody (Zs-BIO) was then diluted with secondary antibody dilution at 1:20,000 and incubated at room temperature for 2 h. The protein was detected using an ECL luminescence kit (Thermo Fisher Scientific, MA, USA), and the protein expression was determined by film strip analysis using ImageJ software.

### Statistical analysis

The data were presented as mean ± standard deviation. One-way analysis of variance was used to compare multiple groups, while the least significant difference method was employed for two-way comparisons between multiple groups. Nonparametric tests were conducted in cases where normality and chi-squareness were not met. A *P* value < 0.05 indicated a statistically significant difference. The statistical analysis was carried out using SPSS 25 statistical software, and GraphPad Prism 8.0 software was used for plotting the data.

## Results

### Effect of non-weight-bearing exercise on pathological changes and Mankin scores in the knee articular cartilage of papain-induced rats with KOA

**Fig. 1:**
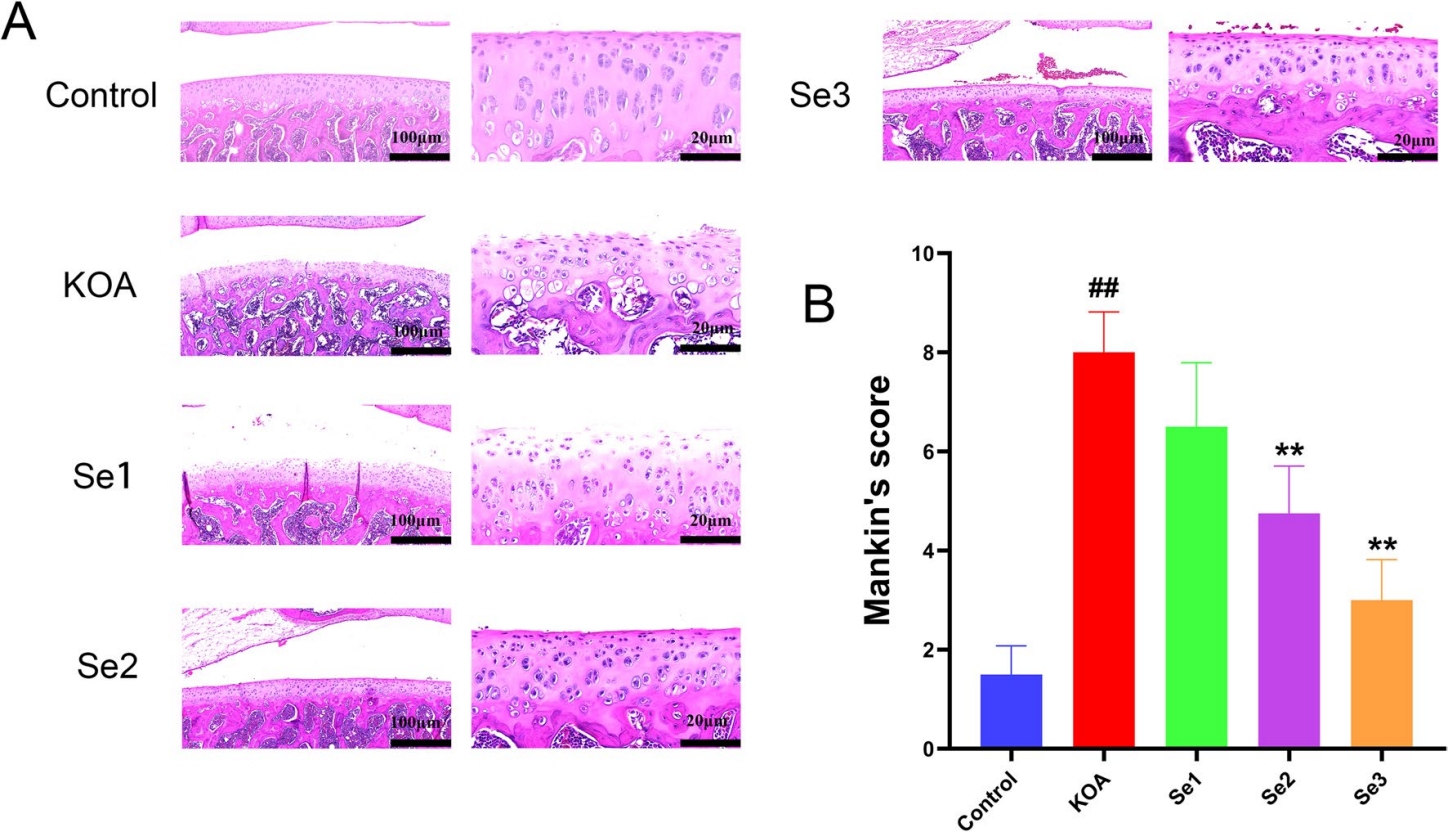
Effect of non-weight-bearing exercise on knee joint pathology in papain-induced rats with KOA. **A** Pathological features of the knee joint of rats with KOA observed after H&E staining. Scale bar = 100 and 20 μm. **B** Mankin score of cartilage tissue of knee joint in rats with KOA. ^##^*P* < 0.01 compared with the control group; **P < 0.01 compared with the KOA group

Injecting papain into the joint cavity led to the rapid development of knee arthritis. The control group showed normal cartilage thickness, a smooth cartilage surface, normal cartilage cell morphology, evenly arranged columnar cells, and a clear tide line. In contrast, the H&E slices in the KOA group were severely damaged with thinning cartilage, defects were visible on the surface of the cartilage, cartilage cell arrangement was significantly reduced and disordered, and tide lines were fractured. In the Se1 group, the joint cartilage thickness became thinner and exhibited an uneven cartilage surface. This group displayed an increase in the number of surface cartilage cells compared with the control group, with cells arranged in a disorderly manner and evident fractures in tide lines. The Se2 group also showed a thinner joint cartilage thickness, but with a relatively smooth cartilage surface. The Se2 group displayed an increase in the number of surface cartilage cells compared with the control group, with some cells arranged in a disorderly manner and evident fractures in partial tide lines. In the Se3 group, the joint cartilage thickness tended to be normal, with a relatively smooth and continuous surface, overall normal cartilage cell morphology, columnar arrangement, and a relatively clear and intact tide line, with some tide lines slightly blurred (Fig. 1A).

Additionally, Mankin scoring was performed on cartilage tissues to assess the severity of cartilage degeneration (Fig. 1B). The control group demonstrated the lowest Mankin scores. In contrast, the KOA group had the highest scores. All three treatment groups exhibited Mankin scores significantly lower than those in the KOA group. This indicated that non-weight-bearing exercise played a significant role in the recuperation of KOA.

### Effect of non-weight-bearing exercise on inflammation in papain-induced rats with KOA

**Fig. 2:**
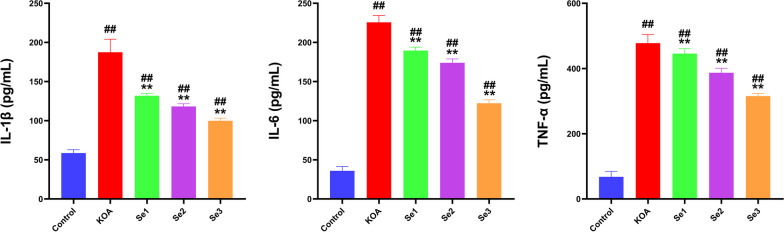
Effect of non-weight-bearing exercise on inflammatory factors in the knee joint of papain-induced rats with KOA. Detection of the expression of inflammatory factors IL-1β, IL-6, and TNF-α in the serum of rats with KOA using ELISA. ^##^*P* < 0.01 compared with the control group; **P < 0.01 compared with the KOA group

As shown in Fig. 2, The serum expression levels of IL-1β, IL-6, and TNF-α were significantly higher in the KOA and Se1-3 groups compared with the control group (*P* < 0.01). Conversely, the Se1, Se2, and Se3 groups displayed a decrease in the expression levels of these three inflammatory factors compared with the KOA group (*P* < 0.01). The Se3 group exhibited the most pronounced drop in the levels of the aforementioned inflammatory factors in the serum. These outcomes suggested that non-weight-bearing exercise could alleviate the inflammatory response associated with KOA.

### Effect of non-weight-bearing exercise on TLR4/MyD88/NF-κB signaling pathway in papain-induced rats with KOA

**Fig. 3:**
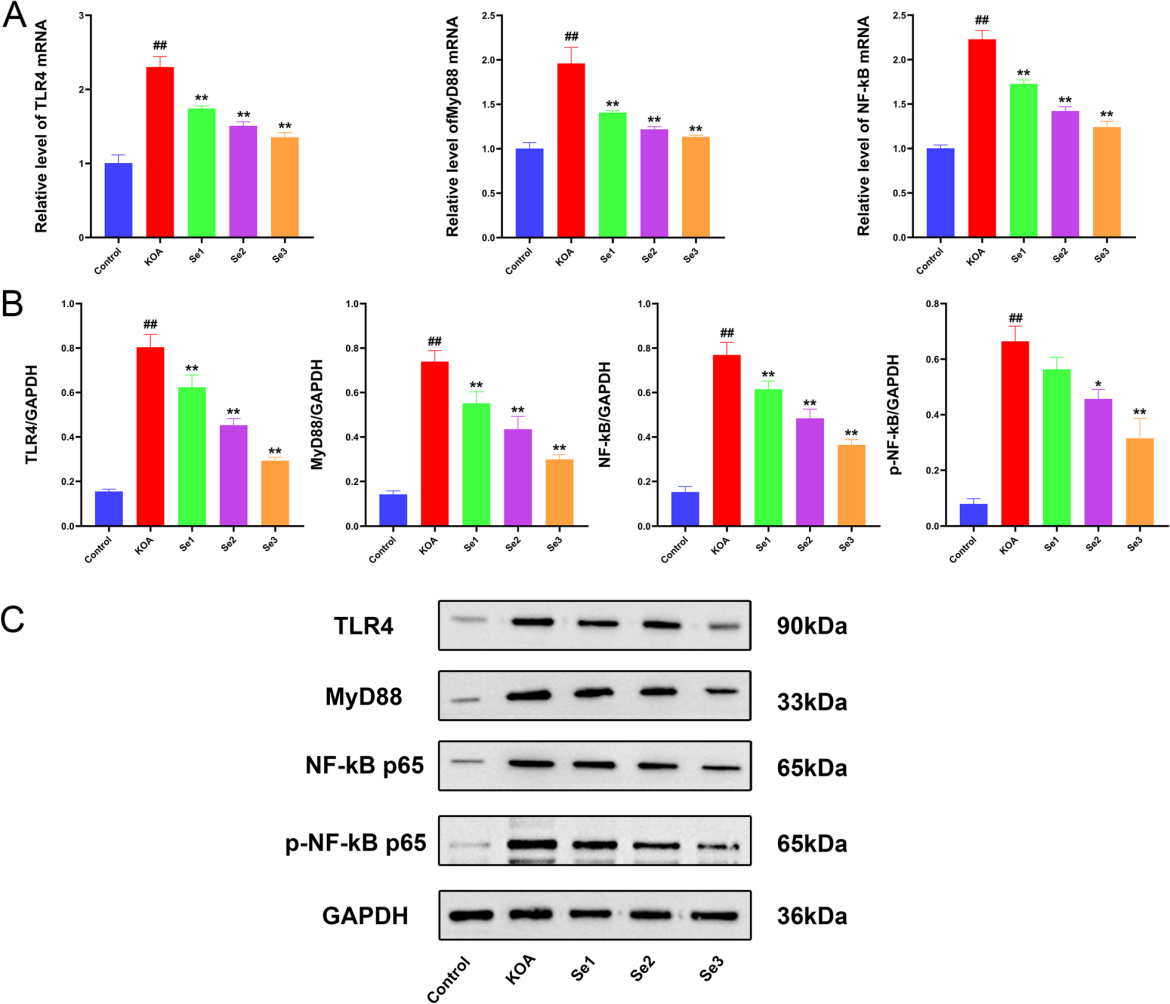
Effect of non-weight-bearing exercise on the TLR4/MyD88/NF-κB signaling pathway in papain-induced rats with KOA. **A** mRNA expression of TLR4, MyD88, and NF-κB was evaluated in rat knee cartilage tissue using RT-qPCR. **B** Protein expression of TLR4, MyD88, NF-κB, and p-NF-κB was evaluated in cartilage tissue using Western blot. ^##^*P* < 0.01 compared with the control group; *P < 0.05 and **P < 0.01 compared with the KOA group

We used RT-qPCR and Western blot to evaluate the changes in the TLR4/MyD88/NF-κB signaling pathway. First, we determined the relative expression levels of TLR4, MyD88, and NF-κB mRNA in rat knee articular cartilage tissue using RT-qPCR (Fig. 3A). The gene expression levels were significantly upregulated in the KOA group. In contrast, the mRNA expression levels of TLR4, MyD88, and NF-κB in cartilage tissue were downregulated in the Se1, Se2, and Se3 groups. Overall, the gene expression levels were most significantly downregulated in the Se3 group. Then, we evaluated the protein expression levels of TLR4, MyD88, NF-κB, and p-NF-κB in cartilage tissue using Western blot (Fig. 3B and C). The results revealed that the protein expression levels significantly increased in the KOA group. However, treating three non-weight-bearing exercises inhibited the expression of related proteins in cartilage tissue, with the most significant effect on the Se3 group. This suggested that non-weight-bearing exercise had a certain inhibitory effect on the TLR4/MyD88/NF-κB signaling pathway in the knee articular cartilage of rats with KOA.

## Discussion

KOA, a multifactorial degenerative disease, leads to symptoms such as pain and loss of function. Early diagnosis primarily depends on a patient's symptoms, signs, imaging examinations, and routine laboratory tests. Modern medical treatments for KOA include exercise therapy, oral Western medicine, and surgery [15–17]. Exercise therapy, including non-weight-bearing exercises, seeks to alleviate clinical KOA symptoms. Surgery is considered when the condition worsens. In the present study, the intra-articular injection of papain, a protein hydrolase [18] that degrades proteoglycans in cartilage, subsequently releases chondroitin sulfate from the matrix, and produces inflammatory factors, was used to establish a KOA rat model that could induce KOA symptoms. Recent studies demonstrated that the intra-articular administration of papain generated KOA symptoms in rats, which were similar to those observed in human KOA [19]. The injection of papain also substantially increased the levels of inflammatory mediators in the serum [20]. As anticipated, this experiment revealed a notable increase in the levels of inflammatory cytokines, such as IL-1β, IL-6, and TNF-α, in rats with KOA. Concurrently, a marked upregulation in the expression levels of TLR4, MyD88, and NF-κB-related genes and proteins was observed.

IL-1β is considered to be a key factor contributing to the development of KOA [21]. It can induce inflammatory responses and chondrolysis independently or exert inflammatory effects together with other mediators. Elevated levels of IL-1β can be found in the synovial fluid, synovium, cartilage, and subchondral layer of patients with KOA. The severity of KOA can be effectively alleviated by inhibiting IL-β. TNF-α, like IL-1β, is considered an essential inflammatory factor associated with the development of KOA, and plays a pivotal role in the degradation of cartilage matrix and bone destruction in osteoarthritis [22]. TNF-α can stimulate the blood vessels surrounding the synovium, resulting in atrophy of the synovial glands. This can indirectly impact the incidence of KOA. At the same time, IL-6 is also an essential inflammatory cytokine in KOA, as a prolonged imbalance of IL-6 can result in the onset and progression of various autoimmune and chronic inflammatory diseases [23].

TLR4, which is an upstream target of NF-κB, has been demonstrated to promote the KOA process through its mediated innate immune responses in chondrocytes, synoviocytes, and osteoblasts [24]. The TLR4/MyD88/NF-κB signaling pathway has been identified in both human and rat articular chondrocytes [25, 26]. Previous chondrocyte experiments [27] revealed that the activation of the TLR4 signaling pathway in human and animal chondrocytes further activated the downstream NF-κB signaling pathway, and p-NF-κB was the main form of NF-κB activation [28]. This led to the release of inflammatory factors such as IL-1β, IL-6, and TNF-α. Therefore, inhibiting the activation of TLR4 and downstream signaling pathways in chondrocytes [29] could reduce the release of corresponding inflammatory factors and slow down the process of cartilage degeneration and KOA. Studies reported that the inhibition of the TLR4/MyD88/NF-κB signaling pathway by gossypol ameliorated IL-1β-induced chondrocyte apoptosis and inflammation [30]. In the rat KOA model, repairing the medial collateral ligament could effectively inhibit the TLR4/MyD88/NF-κB signaling pathway. This led to a decrease in the expression of various inflammatory factors, including IL-6, IL-1β, and TNF-α, ultimately resulting in an improvement in cartilage morphology and a delay in the onset and progression of KOA. Therefore, the TLR4/MyD88/NF-κB signaling pathway represented a promising target for exploring the pathogenesis and treatment mechanisms of KOA.

In the present study, suspension exercise was chosen as the intervention, the suspension method was modified and innovated, and the rats were appropriately rested. Previous studies reported that load reduction by suspension of the hind limbs of rats could delay the progression of KOA [31], but they neglected the fact that rats underwent active or passive knee flexion during the suspension. This caused the generation of mechanical stresses in the articular cartilage. Moderate mechanical stress (e.g., motion) had a protective effect on cartilage and conversely exacerbated KOA [32]. The study examined the underlying mechanism of non-weight-bearing exercise as a treatment for KOA in rats by inducing them to perform knee exercises after suspension. Three different exercise intensities were compared. The study found that non-weight-bearing exercise brought about positive effects, including improved knee joint histological images, alleviated joint cartilage damage, better Mankin scores, reduced levels of inflammatory factors such as IL-1β, IL-6, and TNF-α, and downregulated expression levels of TLR4, MyD88, NF-κB-related genes, and proteins in rats with KOA. The impact of treatment on KOA became more noticeable with the increase in exercise intensity. However, this study had a limitation as it did not examine higher exercise intensities, raising the question of whether excessive mechanical stress worsened KOA. Higher exercise intensities can be explored in further studies.

## Conclusions

This study revealed that non-weight-bearing exercise could effectively mitigate joint cartilage degeneration caused by papain in the rat model of KOA. This was accomplished by diminishing the expression levels of pro-inflammatory cytokine IL-1β, IL-6, and TNF-α in serum, thereby mitigating inflammation and forestalling the onset and advancement of KOA. The underlying mechanism of action might be associated with the TLR4/MyD88/NF-κB signaling pathway.

## Data Availability

The datasets used and analyzed in this study are available with the corresponding author and can be provided on a reasonable request.

## Abbreviations

H&E: Hematoxylin and eosin
IL-1β: Interleukin-1β
IL-6: Interleukin-6
KOA: Knee osteoarthritis
MyD88: Myeloid differentiation factor 88
NF-κB: Nuclear factor kappa B
Se: Suspension exercise
TLR4: Toll-like receptor 4
TNF-α: Tumor necrosis factor-α
